# Multimorbidity in General Practice: Unmet Care Needs From a Patient Perspective

**DOI:** 10.3389/fmed.2020.530085

**Published:** 2020-12-22

**Authors:** Lisanne M. Rimmelzwaan, Mieke J. L. Bogerd, Bregitta M. A. Schumacher, Pauline Slottje, Hein P. J. Van Hout, Marcel E. Reinders

**Affiliations:** Department of General Practice, Amsterdam Public Health Research Institute, Amsterdam University Medical Center, Vrije Universiteit Amsterdam, Amsterdam, Netherlands

**Keywords:** chronic care, multimorbidity, continuity of care, care needs, person-centered care

## Abstract

**Introduction:** In the Netherlands, as in many other countries, current clinical guidelines are directed at single diseases. Patients with multiple chronic conditions may benefit from a more patient-tailored approach. Therefore, our objective is to explore the general practice care needs of patients with multimorbidity from a patient perspective. We also assessed their care experiences and the impact of chronic conditions on their daily functioning.

**Methods:** We conducted a qualitative study, using semi-structured interviews complemented with self-report questionnaire assessments for triangulation, with consenting community-dwelling patients with three or more chronic conditions. Participants were identified through purposeful sampling in three general practices. Two researchers independently coded and thematically analyzed the audiotaped and anonymously transcribed interviews using the constant comparative method. The self-report questionnaire assessments were used to describe the patient characteristics and for triangulation of the data retrieved from the semi-structured interviews.

**Results:** After 12 interviews, saturation was achieved. Overall, most participants were positive about their relationship with the general practitioner (GP) and practice nurse (PN) as well as the care they received in general practice. However, several unmet care need themes were observed: firstly, lack of a holistic approach (by the GP and PN), in particular, insufficient attention to the patient's state of functioning, their limitations in daily life, and their well-being; secondly, they mentioned that personal continuity of care was important to them and sometimes lacking; thirdly, lack of patient-tailored explanations about diseases and treatments.

**Conclusion:** From a community-dwelling multimorbid patient perspective, general practice care could benefit from improving personal continuity of care, attention to personal circumstances and daily functioning, and patient-tailored communication.

## Introduction

The number of patients with multiple chronic conditions, i.e., multimorbidity, increases due to aging of the population and improvements in medical care (1). Patients with chronic conditions experience problems in multiple health areas and when using different health services. Besides physical issues, there are often psychological, social, and cognitive problems (2). Furthermore, multimorbid patients have a poorer quality of life and higher health care utilization than patients with one chronic disease (3).

In Dutch primary care, structured disease management programs are used for three highly prevalent chronic diseases: cardiovascular diseases, diabetes mellitus, and chronic obstructive pulmonary disease (COPD). The goals of these protocolized programs are to provide selective prevention and manage the chronic conditions (4). Within these programs, the general practitioner (GP), practice nurse (PN), and other primary healthcare providers closely collaborate. In the Netherlands, nearly all non-institutionalized citizens are enlisted at one particular general practice. The majority of the routine checkups are performed by the PN. These programs are *disease-oriented*, which in case of multimorbid patients may lead to inefficient and ineffective treatments and can possibly be even harmful in terms of complex medication interactions and more hospital admissions (5). Especially in multimorbid patients, it may be more helpful to adopt a more *person-oriented* approach, which takes account of the individual care needs, preferences, and the social context. A better understanding of the care needs of community-dwelling persons with multimorbidity from a patient perspective is essential to improve person-centered general practice care for this group.

In recent literature, different unmet care needs were mentioned, such as time pressure and lack of personal continuity of care. The qualitative study of Schiotz et al. (6) found three important concerns regarding chronic primary care for patients with multimorbidity: firstly, disease-centered rather than patient-centered care; secondly, lack of attention to comorbidities and patient preferences and needs; and lastly, involvement of numerous healthcare providers with limited care coordination.

Therefore, the objective of this study was to explore the care needs, and for this reason, we also assessed care experiences and the impact of chronic conditions on daily functioning from a patient perspective, in particular, the community-dwelling multimorbid patient.

## Methods

### Study Design

This qualitative study with multimorbid patients is based on semi-structured interviews complemented with a comprehensive self-assessment, which was used for triangulation. The participants were identified through purposeful sampling in three general practices in the area surrounding Amsterdam, the Netherlands. GPs were sampled based on running their practice in diverse areas and populations (metropolitan area/rural area).

This study focused on community-dwelling patients with three or more chronic conditions from a list of selected chronic diagnoses (Table 1). We used this criterion because we expected these patients to have a higher disease burden and thus more care needs than patients with two chronic diseases. Moreover, this criterion was applied in earlier studies in a comparable setting on this topic (7–9). All inclusion and exclusion criteria are listed in Box 1. The participating GPs were instructed to select and invite patients with multimorbidity from their practice to participate in the study. The GPs performed a manual search in their electronic patient records system to identify eligible patients. In order to obtain a diverse sample, the GPs were instructed to select patients from two groups: (1) patients regarded to have self-care difficulties and (2) patients considered to be independent. Self-care difficulties were defined as problems in activities of daily living (ADL) or instrumental activities of daily living (IADL). When patients were willing to participate, the GP asked permission to give the patients' contact details to the researchers. Participants gave permission by written informed consent and were free to end participation at any time. Participants completed the comprehensive self-assessment, which was sent by post one week prior to the interview, and subsequently the interview.

**Table 1:** List of included chronic diseases.

**Clusters**	**Diseases**
HIV/Aids	HIV/Aids
Cancer	All malignant cancer types
Bowel disorders	Diverticular disease
	Crohn disease
	Ulcerative colitis
Cardiovascular	Congenital heart disease
	Infectious disease of heart and/or blood vessels
	Acute rheumatoid heart disease
	Non-rheumatic Valvular Heart disease
	Heart failure
	Angina Pectoris
	Acute myocardial infarction
	Atrial fibrillation/flutter
	Hypertension
	Transient ischemic attack (TIA)
	Cerebrovascular accident (CVA)
	Intermittent claudication
	Aneurysm aortae
	Hypercholesterolemia
Musculoskeletal	Fibromyalgia
	Rheumatoid arthritis
	Cox arthrosis
	Gon arthrosis
	Other arthrosis
	Cervical spine syndromes
	Osteoarthritis spondylosis of the spine
	Low back pain with radiation
	Osteoporosis
Neurologic	Multiple sclerosis (MS)
	Parkinson's disease
	Epilepsy
	Migraine
	Cluster headache
	Trigeminal neuralgia
	Other neuropathies
Alcohol abuse	Chronic alcohol abuse
Psychiatric	Sleeping disorder
	Schizophrenia
	Affective psychosis
	Depression
	Anxiety disorder
	Personality disorder
Respiratory	Chronic obstructive pulmonary disease (COPD)
	Asthma
	Chronic bronchitis
Thyroid	Persistent thyroglossal duct/cyst
	Benign neoplasms of thyroid gland
	Hyperthyroidism
	Hypothyroidism
Diabetes Mellitus	Diabetes mellitus type I
	Diabetes mellitus type II
Urinary	Kidney disease
Psoriasis	Psoriasis with methotrexate use
Obesity	Adiposity
Smoking	Tobacco abuse
Eye disease	Macular degeneration

*The following chronic diseases are also included if patients ever had contact with the GP with this diagnosis*.

*HIV/aids*.

*M. Crohn, Ulcerative Colitis*.

*Alcohol abuse*.

*Schizophrenia*.

Box 1 **In and exclusion criteria.**

**Inclusion criteria**

- Aged 18 or older
- ≥3 diagnoses from the list “Chronic diseases” (Table 1)^*^
- Informed consent

**Exclusion criteria**

- Terminally ill
- Mentally handicapped (ICPC-code P85)
- Diagnosed with dementia (ICPC-code P70)
- Severe hearing or visual impairment (ICPC-codes: H86, F94)
- Insufficient command of the Dutch language
- Patients that are already included in another study
^*^This list “chronic diseases” is developed via multiple brainstorm sessions with the group of GPs who participated in the COPILOT study (7) and includes chronic conditions which are considered as “in need of chronic primary care.” Conditions are coded using the International Classification of Primary Care (ICPC); Supplementary Material. Consensus was reached after comparing the developed list with existing lists of chronic conditions (8).

### Data Collection

The interviews were audiotaped, lasted approximately 1 h, and were conducted at the patient's home. They were semi-structured, guided by a topic list that addressed three themes: (1) the impact of chronic conditions on their lives, (2) care needs, and (3) care experiences. During data collection, debriefing of the initial interviews informed and shaped the following interviews, in particular with respect to (unmet) care needs. Moreover, comprehensive self-report questionnaire assessments were collected prior to the interviews and were used for triangulation during the analyses (10). Items were used from the Patient Assessment of Chronic Illness Care (PACIC) measure (11), a measure that captures experiences with chronic care; (2) European Health Literacy Short Survey Questionnaire (HLS-EU-Q16) (12); and (3) interRAI Check-Up Self-Report (CU-SR) assessment on functional health (13).

### Analysis

Interviews were transcribed verbatim; two researchers (LR, BS) coded and analyzed the anonymized interview transcripts independently. The researchers (LR, BS, MB) conducted a thematic analysis of the interviews using the constant comparative method (14). A coding framework was developed from the initial interviews. Through an iterative process involving comparisons across the manuscripts, these codes were organized. For these codes, themes were developed. Coding and categorizing of the interviews were discussed with the other researchers continuously in the process of the analysis. ATLAS.ti software was used. The self-report questionnaire assessments were used to describe the patient characteristics and for triangulation of the data retrieved from the semi-structured interviews (14, 15).

A comprehensive patient self-assessment was used to corroborate the interview findings. The questionnaire was piloted, tested, and adapted. Prior to the interview, all participants completed the self-assessment. Based on the interview findings, items from the self-assessment were selected by the researchers (MB, LR) for triangulation. The selection of items from the assessment was discussed with and approved by the other researchers. In particular, we used methodological triangulation, which involves using more than one kind of method to study a phenomenon, providing confirmation of findings, more comprehensive data, increased validity, and enhanced understanding of studied themes (15–17). Descriptive analysis of these items was undertaken using SPSS (Version 26).

## Results

### Sample

We invited six GPs running a practice in diverse areas and populations: some were located in a relatively deprived metropolitan area and some in a more rural area. Both areas were covered by the three participating GPs. The reason for refusal of the other three was lack of time. The GPs initially selected 14 eligible patients, and 12 of them were willing to participate. Reasons for refusal were lack of time and an unplanned hospitalization. Saturation was reached after 12 interviews, and therefore, no more patients were recruited. All 12 participants filled in the comprehensive self-assessment.

Two participants lived in a relatively deprived metropolitan area, and the other 10 participants lived in a more rural area. Participants were predominantly female (58%), between 47 and 87 years old with an average age of 72.8 years. All but one participant (92%) had cardiovascular disease (e.g., hypertension, hypercholesterolemia) or diabetes mellitus type 2. Other common chronic diseases included musculoskeletal disorders, cancer (no palliative care), and pulmonary disease. An overview of the participant characteristics can be found in Table 2.

**Table 2:** Participant characteristics & results of the selected items from the self-assessment.

**Nr**	**Age (yrs)**	**Sex^a^**	**Chronic disease**	**How satisfied are you with the contacts with your GP in the last 6 months?^b^**	**Self-reported health^c^**	**To what extent do you experience physical complaints?^e^**	**How much impact do your chronic conditions have on your day-to-day live?^b^**	**How difficult was it for you to make a decision about your illness using the information provided by the GP?^d^**	**During the last 6 months I received written information on how I can improve my health^e^**
1	69	M	Hypertension, diabetes mellitus type 2, COPD, decompensation Cordis, lower back pain with radiation, migraine, psoriasis	8	1	4	5	1	2
2	84	F	Hypertension, diabetes mellitus type 2, asthma, breast cancer, osteoarthritis	8	2	8	8	2	0
3	72	M	Hypertension, diabetes mellitus type 2, hypercholesterolemia, osteoarthritis, prostate cancer	7	2	6	1	1	0
4	85	F	Hypertension, hypercholesterolemia, lower back pain with radiation, rectum cancer	3	2	9	8	dk	0
5	58	F	Hypercholesterolemia, other disease peripheral arteries, hypothyroid	8	2	6	6	2	1
6	59	F	M. Crohn, COPD, osteoporosis, osteoarthritis, allergies	10	1	2	1	1	–
7	67	M	Diabetes mellitus type 2, myocardial infarction, rheumatoid arthritis, allergies	7	1	7	1	2	1
8	47	F	Hypercholesterolemia, stroke, hypothyroid, other disease peripheral arteries	10	2	1	0	1	–
9	77	M	Hypertension, diabetes mellitus type 2, myocardial infarction, colon cancer, bladder cancer, prostate cancer	6	-	-	7	1	0
10	82	M	Hypertension, diabetes mellitus type 2, hypercholesterolemia, atrial fibrillation, laryngeal cancer	8	3	5	5	2	–
11	86	F	Atrial fibrillation, stroke, osteoarthritis, osteoporosis, basal-cell carcinoma	-	1	7	1	1	–
12	87	F	Hypertension, stroke, hypothyroid, osteoarthritis, squamous cell carcinoma	8	2	6	4	2	0

a *M, male; F, female*.

b *On a 10 point scale from zero (no) to ten (enormous)*.

c *0, perfect; 1, good; 2, reasonable; 3, bad; 4, could or would not answer*.

d *1, Very easy; 2, Relatively easy; 3, Relatively difficult; 4, Very difficult; dk, Don't know*.

e *0, almost never; 1, generally not; 2, sometimes; 3, most of the time; 4, almost always*.

### Comprehensive Self-Assessment

The selected items from the self-assessment are presented in Table 2 as well.

### Interviews

The themes that were elicited from the interviews in terms of care needs, care experiences, and the impact of chronic conditions on patients' functionality are reported below, illustrated by particular quotes. The results of the selected items from the comprehensive self-assessment are presented at the end of this paragraph and in Table 2.

#### Holistic Approach

Participants found that the checkups for their cardiovascular diseases, diabetes, and/or COPD received from the PN contributed to their health. Some participants perceived they missed an approach that focuses on the patient “as a whole.” For example, participants expressed the need for their GP and PN to pay more attention to their state of functioning, their limitations in daily life, and their well-being. Some participants stated that GPs and PNs seem to focus too much on the use of clinical guidelines that address prevention and management of diseases and that there is too little focus on the functioning and well-being.

“*I found it important that healthcare providers look at patients as a whole. I am having trouble with that protocol thing. People like it when a healthcare provider pays attention to them, in addition to following protocols.”*

“*When I needed painkillers, I had words with my general practitioner about that. I had morphine, but she didn't want to prescribe anymore of them due to the risk of addiction. Pain medication is important for my functioning. She only thought of the addiction.” (P1)*.

#### Personal Continuity of Care

Participants stated that it is important that a primary healthcare professional knows them. They found it annoying to repeat their situation to a care provider who did not know them.

“*I think my general practitioner knows what I am like. I have been his patient for a long time now. He knows me well and that helps in advising me. It would be frustrating if my general practitioner did not know me so well and I had to explain my situation to him all over again.” (P10)*

Participants reported that personal attention is one of the most important aspects of a good therapeutic relationship between primary healthcare providers and patients. According to them, health care providers should treat their patients as equals and take their patients seriously.

“*In my opinion, personal attention is the most important. No one likes to be treated as if they were a number. A doctor may see countless patients on a single day, so I understand that there is a risk of getting lost in a daily routine… However, every patient is different; every patient has a different story to tell.” (P8)*

#### Time Constraints

Participants expressed concerns about the increasing time pressure and personnel shortage in the primary care system.

“*GPs nowadays have less time for their patients than back in the days. Recently, I visited my general practitioner and mentioned that I had two physical complaints. The general practitioner told me that there was only time scheduled for one complaint. In the past, this would have never happened. There is not much the current general practitioners can do about it. It is a consequence of increasing time pressure. However, it is difficult for patients too.” (P2)*

#### Communication Between Primary Healthcare Providers and Patients

About their relationship with the GP, participants were all positive during the interviews. They reported that their GP was reliable, approachable, and concerned with them. The participants felt safe and comfortable with their GP and felt that they could address the issues that they were concerned with, such as diseases or fears.

“*My experiences are good. I see my general practitioner and practice nurse because of my diabetes. I have no complaints. The connection with the doctor is good, I trust him 100%. If I have something, I know I can always call him.” (P3)*

However, participants felt that explanations about diseases or treatments should be more tailored to their level of comprehension. For example, primary healthcare providers sometimes tended to use medical jargon, take too little time to explain matters, or only provided explanations verbally, and did not sufficiently check with the patients if everything is understood:

“*My general practitioner tries to explain everything clearly to me, but he does not write it down. When I get home I have already forgotten what he said. For me, it would be helpful if he would write the treatment plans or explanations down. It is the same when I visit the practice nurse*.

She explains too much. I cannot remember all this information, and it is too theoretical.” (P7)

#### Physical Functioning

Half of the participants mentioned to need some support in ADL or IADL activities (P1, P2, P6, P10, P11, and P12). While some of these participants only needed help with IADL such as doing grocery shopping, there was one participant who needed help with ADL activities. Some of these participants reported problems in their mobility, mostly in walking. However, the other half of the participants did not feel chronically ill and did not experience physical limitations in their daily life.

“*Honestly, I do not feel limited at all in my daily activities. Of course I can feel that I am almost old and no longer 65 but that is just a logical consequence of aging.” (P2)*

“*I am still surprised that I belong to the category of ‘multimorbid patients.’ In my opinion, I am not the prototype of a chronically ill patient.” (P3)*

#### Psychological Functioning

Most participants felt that they could cope with their diseases in a good way most of the time. However, participants reported psychological consequences of their diseases. Fears for new diseases, death, dependency, and embarrassment about the consequences of their diseases were mentioned consistently.

“*I do not want to get old and end up in a nursing home. The thought of having to ask my wife to take off my socks is already terrifying.” (P3)*

#### Social Functioning

##### Support system

As for support from family, friends, and neighbors, the participants were satisfied. All participants had several people around them who could help or already were involved in activities like grocery shopping, driving long distances, and finances. Moreover, a lot of the participants not only were the receiving party but also provided help to others. Two participants were experienced volunteers.

Maintaining social contacts, especially with family, was very important to all the participants. They reported that their social contacts had a big share in their quality of life.

##### Social participation restriction

Even though the participants were satisfied with the support and contact they had with family and friends, some participants noticed that their social life was getting smaller. At times, they felt as if they no longer were seen as a full member of society due to their impairment.

“*Sometimes I felt miserable. Because of my physical limitations, my social life was getting smaller, my work ended, and it felt like I no longer participated in society.” (P5)*

#### Triangulation

Mostly, the findings of the interviews were in line with the results of the self- assessments, such as the self-reported health and the influence of the chronic conditions on their daily lives. Contradictions were also found. Based on the self-assessments, participants seem very positive about different topics, whereas in the interviews, points of improvements on those same topics are mentioned. For example, the decision-making process regarding their chronic conditions based on the provided information by the GP was assessed. In the self-assessment, the majority of the participants experienced the decision-making process “without difficulty.” In the interviews, however, multiple participants mentioned that explanations about diseases and treatments tailored to the patients' level of comprehension and preferred mode during the decision-making process were needed (Table 2).

## Discussion

These findings contribute to our understanding of the care needs, care experiences, and the impact of chronic conditions on daily functioning of community-dwelling patients with multimorbidity.

In this study, participants expressed the need for a holistic approach. This is in line with our expectation that patients with chronic conditions and especially patients with multiple chronic conditions are in need of more attention to their contexts and preferences. On a similar note, patients experience a lack of focus on patients' functioning in the current structured disease management programs. In line with our findings, Noel et al. (18) found that patients were more concerned about their functioning and the way their diseases interfere with their lives than the symptoms *per se*. Moreover, Huber et al. (19) also found a discrepancy in impact of daily functioning on health between doctors and patients. Building upon the previously mentioned and our own findings, we postulate that GPs possibly prioritize care decisions based on disease management and long-term health risks, in contrast with patients, who prioritize care needs based on their functioning (short-term health gain).

The need for personal continuity of care emerged from the interviews. This result is consistent with those of other studies (20, 21). Schiotz et al. (6) reported that patients with multimorbidity are most likely to gain from continuity of care since they have a high treatment burden in terms of understanding and self-managing their conditions; they attend multiple appointments and sometimes manage complex drug regimens.

Concerning the care needs in the communication between the GP or PN and patient, participants mentioned that explanations about diseases or treatments should be more tailored to their level of comprehension and preferred mode (oral or written information). These findings are in line with previous findings (21–24). Regarding the impact of multimorbidity on functioning, most of the participants did not consider themselves chronically ill, despite having three or more chronic conditions. The participating patients appeared to accept their limitations and adapted their lives to it. Self-reliance, having social contact, and being mobile were important factors. This is in line with the newly proposed definition of health, which focuses more on the patients' ability to adapt and to self-manage (19). On the other hand, participants consistently mentioned fears for future dependency due to functional loss, and they were aware that they were living in a fragile system.

Most of the identified unmet needs with respect to general practice care may not be unique to patients with multimorbidity only. However, we think multimorbidity increases the probability that these needs are unmet. Building upon this, we suggest that multimorbidity magnifies the importance of the mentioned care needs.

### Strengths and Limitations of This Study

Firstly, in this qualitative study, we triangulated our results by comparison with the self-assessments in order to strengthen the validity of our findings. Mostly the findings of the interviews were in line with the results of the self-assessments, such as the self-reported health and the influence of the chronic conditions on their daily lives. Secondly, different researchers were involved in the data analysis, which strengthens the interpretation of our findings in terms of objectivity. Thirdly, this study utilized purposive sampling to enable variation of our sample within the multimorbid population (three or more chronic conditions) in terms of (in)dependence in self-care and by selecting general practices in metropolitan and rural areas.

Some limitations can be identified. Firstly, although the GPs served diverse populations, only three GPs participated in our study. This limits the ability to address differences in GP–patient interactions and care experiences of the patients. All were experienced GPs (>10 years work experience). The three GPs had a comparable number of patients in their practices. Secondly, more importantly, possible selection bias might have occurred in patients who had no trouble with expressing themselves and their needs during an interview and in the self-report questionnaire or patients who were on good speaking terms with their GP. We remain uncertain whether and how needs and care experiences of patients from non-Dutch-speaking persons or persons who are less familiar with their GP might differ.

### Implication for General Practice and Future Research

Despite these limitations, our study *results have some practical implications for improving care for patients with multimorbidity. Our findings suggest that in primary care, there is a need for more focus on context and functioning in care for patients with multimorbidity*. Better incorporation into the vocational training might be beneficial. Lastly, to improve continuity of care (especially relational continuity) in general practice, more research and practical solution on this important topic are needed.

## Conclusion

This study explored unmet general practice care needs of community-dwelling multimorbid patients in the Netherlands. Overall, the current disease-oriented chronic care programs do have their benefits. However, there is a need for better personal continuity of care, patient-tailored communication, and more focus on patient's context and functioning instead of disease management alone.

## Data Availability Statement

The datasets generated for this study are available on request to the corresponding author.

## Ethics Statement

The studies involving human participants were reviewed and approved by ethical clearance was granted by the VU University medical center's ethical committee. The patients/participants provided their written informed consent to participate in this study. Written informed consent was obtained from the individual(s) for the publication of any potentially identifiable images or data included in this article.

## Author Contributions

MR obtained the funding and coordinates the study. LR, HV, and MR were involved in the study design. LR, MB, BS, HV, and MR were involved in the implementation of the study. LR, BS, and MB wrote the first substantial draft of the article and were guarantors. All authors critically revised the manuscript, read, and approved the final manuscript.

## Conflict of Interest

The authors declare that the research was conducted in the absence of any commercial or financial relationships that could be construed as a potential conflict of interest.
